# Incidental detection of a lung perfusion defect suspicious of pulmonary embolism on labelled white blood cell quality control imaging—case report

**DOI:** 10.1186/s43055-021-00475-4

**Published:** 2021-04-01

**Authors:** Osayande Evbuomwan, Gerrit Engelbrecht

**Affiliations:** Nuclear Medicine Department, Universitas Academic Hospital, University of The Free State, Lower ground floor, Logeman Street, Bloemfontein, 9301 South Africa

**Keywords:** Quality control, White blood cell labelling, Perfusion defect, Pulmonary embolism, Case report

## Abstract

**Background:**

Incidental findings could be a very important observation in various nuclear medicine studies. There have been few cases of incidental findings of perfusion abnormalities on early quality control images of the lungs during radiolabeled white blood cell studies. This study is the first to detect perfusion defects on the early quality control images of the lungs during a labelled white blood cell study suspicious of pulmonary embolism in an unknown but treated COVID-19 patient.

**Case presentation:**

We present a 40-year-old male who was referred to our department for a nuclear medicine ^99m^Tc HMPAO-labelled white blood cell study to rule out osteomyelitis of his right foot. Early 5-min quality control images of his lungs revealed two perfusion defects in the right lung. A suspicion of pulmonary embolism was made, and a perfusion only SPECT/CT study the next day confirmed the suspicion of pulmonary embolism in one of the defects, with a possible fissure sign as a differential.

**Conclusion:**

There has been an increase in the incidence of lung perfusion abnormalities and pulmonary embolism during the COVID-19 pandemic. Some of these may be detected as incidental findings on early lung quality control images of radiolabeled white blood cell studies.

## Background

Scintigraphy with autologous labelled white blood cells (WBCs) is a widely used method to detect sites of infection. There are various quality control (QC) methods used to assess the quality of WBC labelling [1]. Early images of radiotracer distribution in the lungs are one of them [1, 2], and this is routinely performed in our facility. Incidental findings could be a very important observation in various nuclear medicine studies. These findings can be identified during imaging, processing or reporting of these studies. In the era of the coronavirus disease 2019 (COVID-19) pandemic, there have been several reports on lung perfusion abnormalities associated with the disease [3–6]. In this case report, we highlight an incidental finding of lung perfusion defects suspicious for pulmonary embolism (PE), identified on early technetium 99 metastable hexamethylpropyleneamine oxime (^99m^Tc HMPAO)-labelled WBC QC images of the lungs of a patient unknown to have recently recovered from a previous COVID-19 infection. There have been several documentations in the past revealing incidental detection of abnormalities on these early lung QC images [7, 8]. However, we were not able to see any that had categorically identified lung perfusion defects suspicious for PE.

## Case presentation

**Fig. 1:**
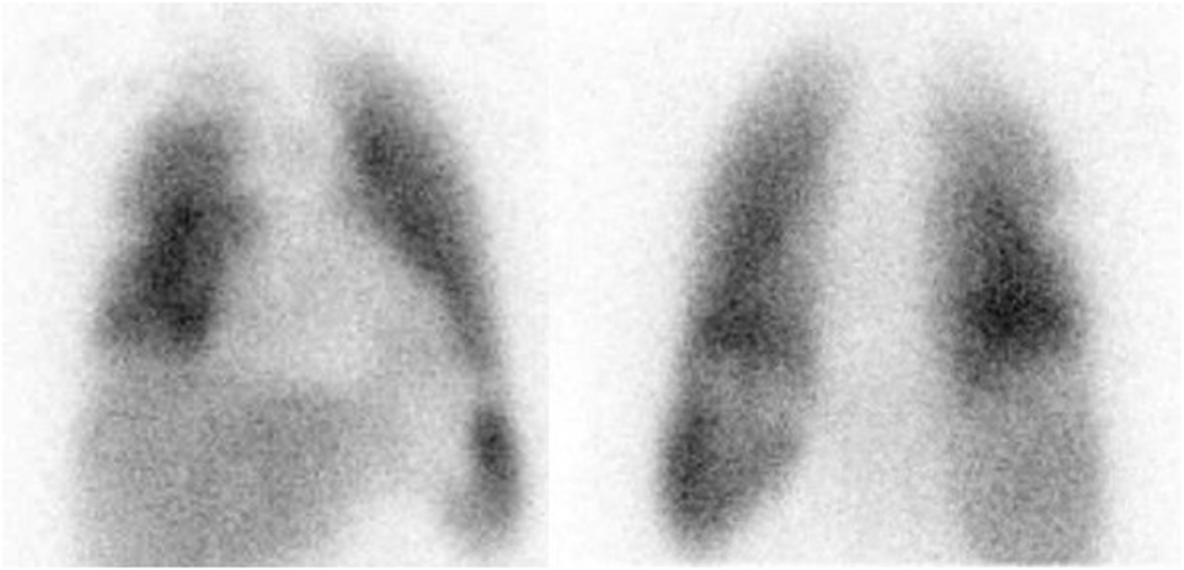
Anterior and posterior images of the lungs (left and right respectively) 5 min after injecting 25mCi of technetium 99m-labelled white blood cells

**Fig. 2:**
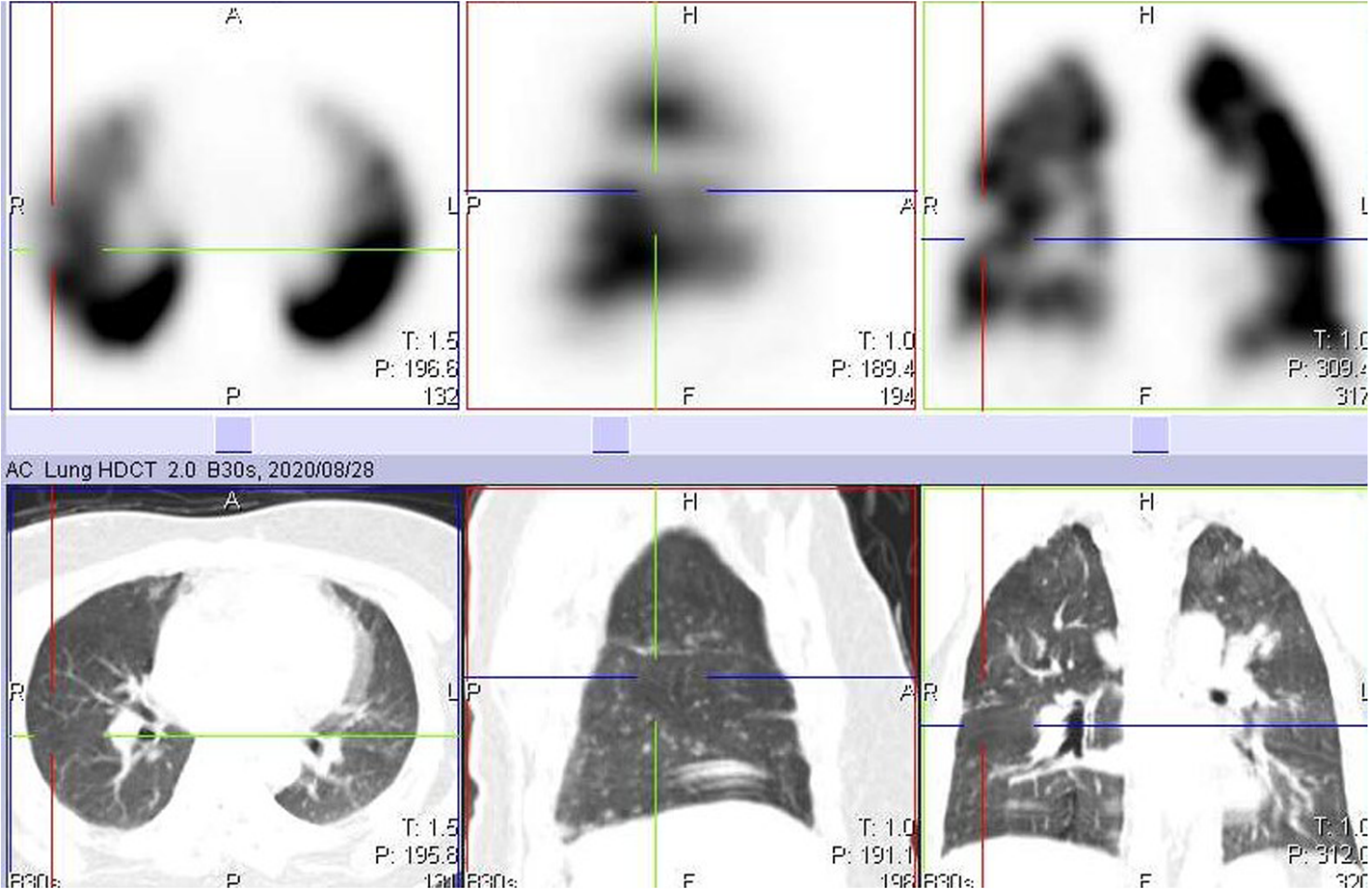
Axial, coronal and sagittal SPECT images of the lungs showing a large perfusion defect in the lateral segment of the right middle lobe. The corresponding CT images below do not show obvious structural lung changes in that segment

We present a 40-year-old male who was referred to our facility for labelled WBC imaging to rule out osteomyelitis in his right foot. Early quality control images of his lungs revealed perfusion defects in the lateral and apical segments of his right middle and upper lobes respectively (Fig. 1). After reviewing these images, we informed the referral doctors of a possible suspicion for PE, and thus, a lung perfusion single-photon emission computed tomography/computed tomography (SPECT/CT) study with ^99m^Tc macroaggregated albumin (MAA) was booked the next day. We could not perform lung SPECT/CT imaging of the labelled WBC study because we reviewed those early images over an hour after injection of the radiotracer and, by then, clearance of the normal physiological uptake of the radiotracer in the lungs had occurred. The ^99m^Tc MAA lung perfusion only SPECT/CT study revealed a perfusion defect in the lateral segment of the right middle lobe, although close to the major fissure, we could not totally rule out PE (Fig. 2). Unfortunately, we could not confirm if this defect is matched or unmatched on ventilation images, as were not performing lung ventilation studies then, and thus, we reported it as a non-diagnostic finding for PE. It was part of our departmental protocol then not to perform ventilation studies because of the potential increased risk of spread of COVID-19 infection.

**Fig. 3:**
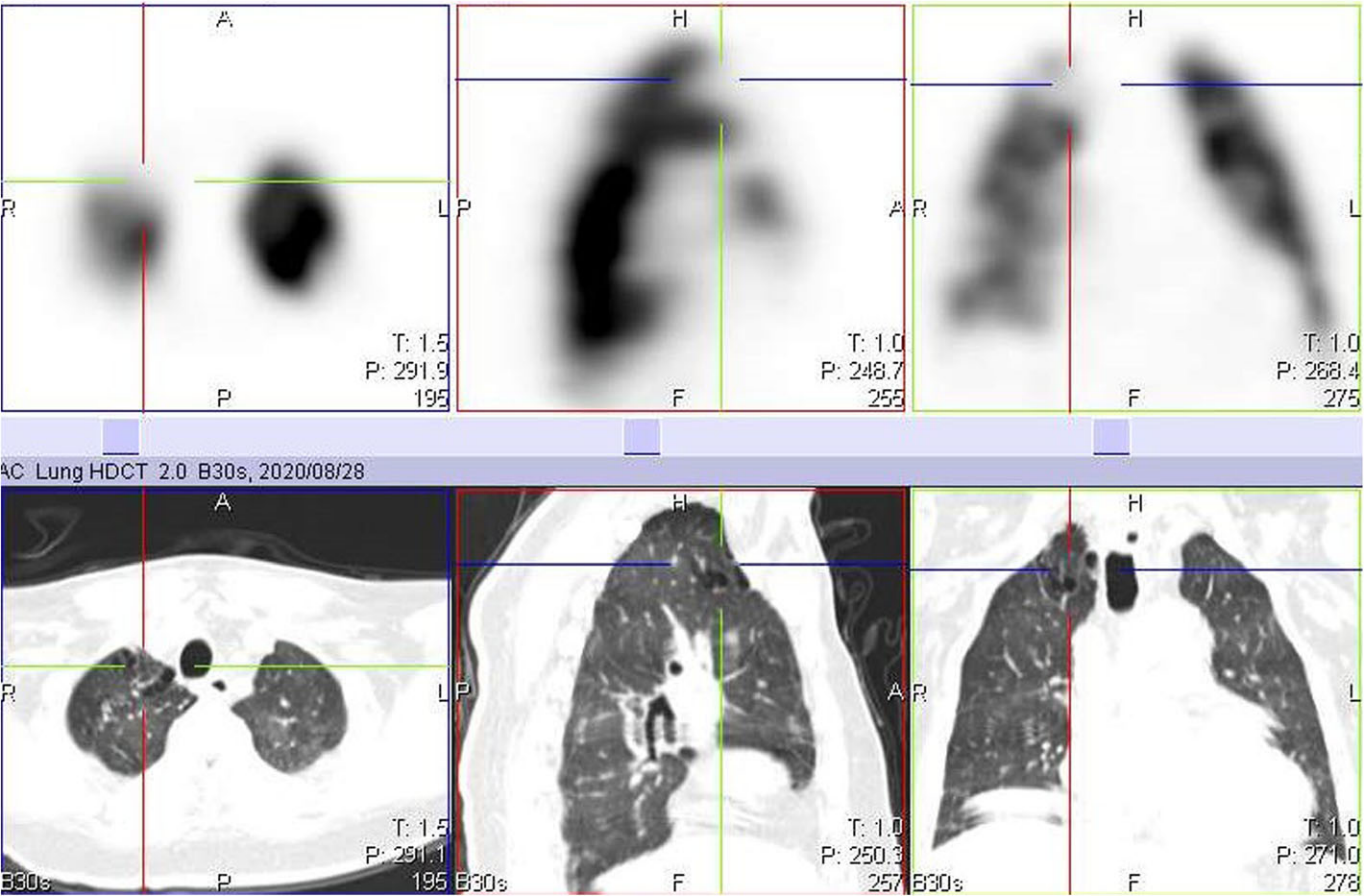
Axial, coronal and sagittal SPECT images of the lungs showing another large perfusion defect in the apical segment of the right upper lobe. However, the corresponding CT images below show structural lung changes with mosaic attenuation in that segment

A second defect in the anterior segment of the right upper lobe was also demonstrated on the ^99m^Tc MAA lung SPECT/CT images, corresponding to mosaic attenuation changes on CT (Fig. 3). Mosaic attenuation, although not specific, has also been described in the literature to be associated with perfusion abnormalities in COVID-19 infection [9, 10].

**Fig. 4:**
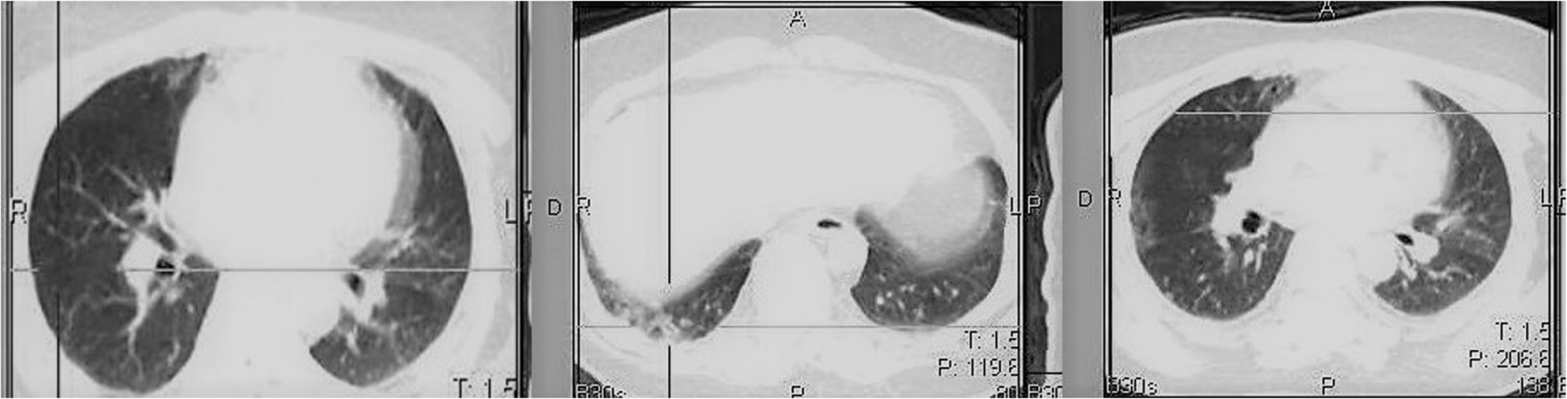
Axial CT images in different slices showing ground glass opacities in the right lung on the left and right images, with the middle image showing linear consolidation in the right lung. Note the predominant peripheral distribution

Incidentally, on the CT images, we identified ground glass opacities and linear consolidation in the right lung, with a predominant peripheral distribution (Fig. 4). These radiological findings on lung CT have been well documented to be associated with COVID-19 infection [9, 11].

This finding, alongside the suspicion for pulmonary embolism and the mosaic attenuation on CT, raised a suspicion for an ongoing or previous COVID-19 infection. Further investigation and clinical history revealed that the patient had just recovered from COVID-19 infection 2 weeks prior to his referral to our department.

## Conclusion

This case is a very good case for teaching and serves as a reminder that incidental findings in imaging must always be looked out for, besides the primary reason for an imaging study. Quality control imaging of the lungs during radiolabeled WBC studies might not be routinely done in most nuclear medicine centres; however, incidental findings such as those noticed in our study could be missed. We are not sure what the clinical significance of these defects may mean for the patient later on; however, we informed the referral doctor accordingly.

## Data Availability

All data and material of the article are readily available.

## Abbreviations

WBC: White blood cell
QC: Quality control
COVID-19: Coronavirus disease 2019
PE: Pulmonary embolism
^99m^Tc HMPAO: Technetium 99 metastable hexamethylpropyleneamine oxime
SPECT/CT: Single-photon emission computed tomography/computed tomography
MAA: Macroaggregated albumin
